# Sestrin2 as a Potential Target in Hypertension

**DOI:** 10.3390/diagnostics13142374

**Published:** 2023-07-14

**Authors:** Steven Didik, Hao Wang, Adewale Segun James, Lily Slotabec, Ji Li

**Affiliations:** 1 Department of Surgery, University of South Florida, Tampa, FL 33612, USA; sdidik@usf.edu (S.D.); wangh@usf.edu (H.W.); adewalesegunjames@usf.edu (A.S.J.); lslotabec@usf.edu (L.S.); 2 James A. Haley Veterans’ Hospital, Tampa, FL 33612, USA; 3 Department of Physiology and Biophysics, Mississippi Center for Heart Research, University of Mississippi Medical Center, Jackson, MS 39216, USA

**Keywords:** Sesn2, redox homeostasis, signal transduction

## Abstract

Hypertension is a highly complex, intricate condition affecting millions of individuals across the globe. Nearly half of adults in the United States are diagnosed with hypertension, with incident rates projected to rise over the next decade. Hypertension is a precursor to many cardiovascular diseases including atherosclerosis, stroke, myocardial infarction, heart failure, and peripheral artery disease. This review describes the major processes contributing to the development of hypertension and how Sestrin2 (Sesn2), an antioxidative protein, could be a potential target in the treatment of hypertension. In hypertension, increased reactive oxygen species (ROS) production is a critical component in the etiology of the condition. The increased ROS in hypertension is derived from a variety of sources, all of which are covered in depth in this review. Increased ROS is generated from mitochondrial stress, endoplasmic reticulum (ER) stress, NADPH oxidase (NOX) overactivity, and the uncoupling of endothelial nitric oxidase synthase (eNOS). Sesn2, a highly conserved, stress-inducible protein, has the structural and functional characteristics to be a potential therapeutic target to alleviate the progression of hypertension. The structure, function, genetics, and characteristics of Sesn2 are presented in the review. The Nrf2/Sesn2, Sesn2/AMPK/mTOR, and Sesn2/Angiotensin II signaling pathways are described in detail in this review. Sesn2 can be utilized in a multitude of ways as a therapeutic modality in hypertension. This review explores potential Sesn2 inducers and activators and how Sesn2 can be incorporated into gene therapy for the treatment of hypertension.

## 1. Introduction

Hypertension is characterized by continual elevated blood pressure exerted systemically in the arteries [1]. The condition is intricate and vastly complex, derived from numerous interconnected etiologies, contributing to the challenge of developing novel treatments [2]. There are several genetic and environmental factors that contribute to the development of hypertension. It is estimated that almost half of adults in the United States had a diagnosis of hypertension in the years 2015–2018, with hypertension being uncontrolled in 79% of cases [3]. Estimated yearly medical costs of hypertension have been recorded at $79 billion (2016) and estimated hypertension-associated lost productivity costs have been reported at 23.6 billion (2008) [3].

Hypertension is a major risk factor for a myriad of cardiovascular diseases such as stroke, myocardial infarction, atherosclerosis, heart failure, and peripheral artery disease. Hypertension can be divided into two groups: essential and secondary hypertension. Essential hypertension is idiopathic, caused primarily by genetics and environmental stressors [4]. Essential hypertension constitutes almost all hypertension diagnoses [4]. Secondary hypertension constitutes roughly 5% of all hypertension diagnoses and originates from diseased organ and bodily states [5]. Dysfunction of vital organs, including the kidneys and vasculature, can contribute to the development of secondary hypertension [5].

Hypertension can be classified as normal, pre-hypertension, stage one, and stage 2 [4]. Normal hypertension includes systolic blood pressure (SBP) at levels > 130 mm and diastolic blood pressure (DBP) levels > 85 mm. Pre-hypertension includes SBP 130–139 mm and DBP 85–89 mm. Stage 1 hypertension includes SBP 160–179 mm and DBP 90–99 mm. Stage 2 hypertension includes SBP 160–179 mm and DBP 100–109 mm [4]. Essential hypertension is modifiable, with changes in diet, activity levels, and stress management proving effective in reducing blood pressure levels.

A multitude of therapeutic interventions for hypertension exist, but due to the confounding nature of the condition, new strategies must be investigated to contribute to the amelioration of the condition. In this review, we will establish a foundational knowledge of the etiology of hypertension from the perspective of the influence of oxidative stress. Following this, we will introduce the structure and function of an exciting protein: Sestrin2 (Sesn2). Lastly, we will intricately link the etiology of hypertension to how Sesn2 could potentially serve as a target in the treatment of hypertension.

## 2. Hypertension Etiology

### 2.1. The Oxidative Stress Approaches

**Figure 1:**
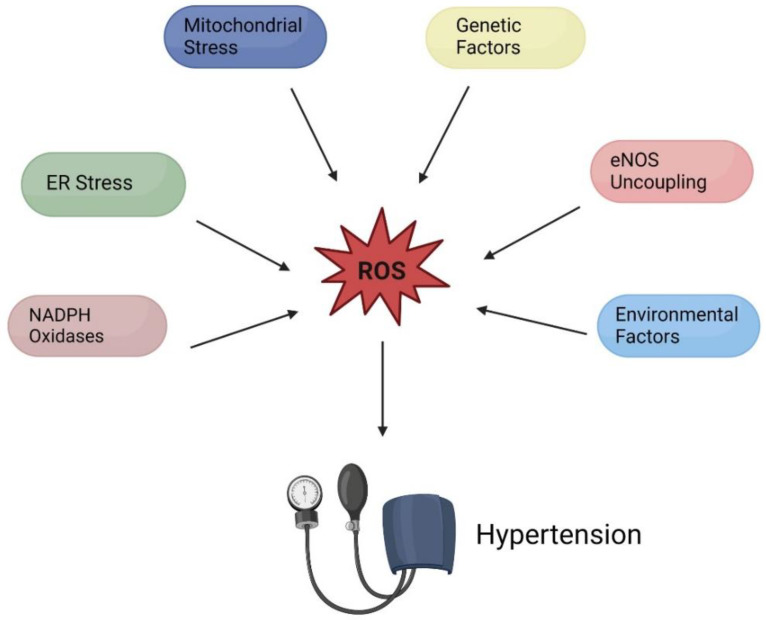
Sources of ROS contributing to the development of hypertension (created with BioRender.com, accessed on 10 May 2023).

Various organ systems, including the vasculature, heart, nervous system, endocrine system kidney, and immune system work simultaneously and make moment-by-moment adjustments to maintain normal blood pressure [5,6]. Intra or inter-misregulations occurring within or between the organ systems can lead to pathological imbalances that contribute to hypertension. Underlying these dysfunctions is a major contributor to the development of the condition: oxidative stress [6]. Increased oxidative stress results in an increase in reactive oxygen species (ROS) [7]. Increased ROS produces detrimental consequences including cell death, cell injury, and DNA damage [6]. It should be noted here that normal levels of ROS are essential for physiological function. Under healthy conditions, ROS serve an array of functions including the oxidative burst exhibited in immune defense, as well as the oxidative post-translational modification of proteins [6,7]. The oxidative post-translational modification of proteins by ROS in normal physiological conditions is reversible, allowing the target proteins to temporarily change function to serve various advantageous roles [6]. In hypertension, excess ROS production leads to irreversible functional changes in proteins, leading to cell death, injury, and organ failure [6]. The misregulation of the redox system in hypertension can be attributed to ROS produced ineffectively by various sources including mitochondrial stress, endoplasmic reticulum (ER) stress, NADPH oxidase (NOX) hyperactivity, and uncoupled endothelial NO synthase (eNOS) (Figure 1) [8].

### 2.2. Mitochondrial Stress Contributing to Hypertension

Mitochondria serve a variety of functions, including ATP generation through oxidative phosphorylation, regulation of redox balance, and the production of ROS [9]. The production of mitochondrial ROS is a complex and regulated process, where superoxide (O2^−^) is produced from electrons being added to oxygen during the electron leak of the electron transport chain [9]. The structural and functional proteins of the mitochondria are sensitive to ROS hyperactivity. The hallmark increase in ROS during hypertensive conditions is generated by a myriad of complex and intricate potential sources. When high levels of ROS react with the mitochondrial structure and mitochondria-associated proteins, these proteins become inactive and structural damage can occur, contributing to mitochondrial dysfunction leading to hypertension [9]. As mitochondrial dysfunction persists, it further exacerbates the severity and progression of hypertension in a feed-forward manner. A structure affected by the feed-forward mechanism of the mitochondrial stress exhibited in hypertension is the main regulator of the vasculature, the endothelium. Under normal conditions, the level of ROS produced by endothelial mitochondria serves an array of functions, most notably, a contribution to effective vascular dilation [10]. Upon the introduction of hypertension-generating risk factors, endothelial mitochondrial ROS is produced in excess and is hyperactive. As a result, pro-inflammatory mediators are activated, and endothelial dysfunction occurs, contributing to the development of hypertension [10]. Based on these mechanisms, decreasing/regulating mitochondrial ROS is a viable target in the treatment of hypertension. Prior studies have shown that superoxide scavengers and mitochondrial antioxidants are effective treatment modalities in attenuating elevations in blood pressure [10]. The development or time-dependent activation of an effective therapeutic agent could potentially diminish the high level of ROS produced by the mitochondria, thus re-establishing the redox imbalance of hypertension.

### 2.3. Endoplasmic Reticulum (ER) Stress Contributing to Hypertension

The endoplasmic reticulum (ER) is a sub-cellular organelle that serves a diverse number of functions ranging from the insurance of proper protein assembly and transport to lipid steroid and carbohydrate synthesis [11]. A regulatory mechanism of the ER is the unfolded protein response. This response is highly regulated, involving a complex network of signaling pathways, and upstream and downstream effectors [12]. The long-term activation of the unfolded protein response is known as ER stress. Causative factors, coupled with increased oxidative stress associated with hypertension can trigger ER stress furthering the progression of the condition. Persistent ER stress is an agent in the etiology of an array of cardiovascular diseases, including hypertension [13]. With chronic ER stress, protein misfolding occurs and, as multiple misfolded proteins amalgamate, cellular dysfunction then ensues, thus promoting further dysfunction and progressing the development of hypertension [11]. Mitochondria and the ER have crosstalk capabilities via ER-associated mitochondrial membranes [12]. Protein production is an energy-dependent process, and thus, due to the abnormally high energy requirement under ER stress conditions, depletes ATP. Mitochondria then begin to produce ATP via oxidative phosphorylation in response and excess ROS is generated [12]. Regulation of ER stress is a pivotal aspect of targeting hypertension. Targeting oxidative stress contributing to overactivation of the unfolded response could potentially restore the unfolded protein response’s activity to homeostatic rates, attenuating the progression of hypertension.

### 2.4. NADPH Oxidase (NOX) Hyperactivity Contributing to Hypertension

Superoxide (O_2_^−^) and H_2_O_2_ are two primary types of ROS of focus in hypertension. They are produced enzymatically by nicotinamide adenine dinucleotide phosphate (NADPH) oxidases (NOXes) [7]. In all studied animal models of hypertension, it has been substantiated that NOX ROS misregulation/overactivity plays a pivotal role in condition development [6]. NADPH oxidase is a membrane-bound multi-subunit enzyme that catalyzes superoxide formation [6,7]. Found primarily in phagocytic cells, there are seven known isoforms: NOX1, NOX2, NOX3, NOX4, NOX5, Duox1, and Duox2. The isoforms are expressed throughout the body with NOX1, NOX2, NOX4, and NOX5 expressed in endothelial cells, smooth muscle cells, fibroblasts, and various immune cells [14]. All isoforms play a critical role in condition development, as well as in the maintenance of normal physiological functions [14]. Of most relevance, NOX1 and NOX2 are involved in the development of hypertension [14]. NOX4 also plays a role in hypertension development at non-physiological, overactive levels. NOX4 serves vasculo-protective purposes under normal physiological conditions, but when overexpressed, NOX4 proves injurious and contributes to condition progression [6,14]. The prohypertensive and inflammatory factors including Angiotensin II (Ang II), Tumor necrosis factor (TNF), aldosterone, growth factors, salt, sources from poor diet, and endothelin 1 (ET-1) are misregulated in hypertension and interact with NADPH oxidases [6]. In turn, the overactivation of NOX occurs, contributing to increased ROS production contributing to the pathophysiology of hypertension [6]. Various NADPH oxidase inhibitors targeting NOX1, NOX2, and NOX4 have been utilized to target these enzymes; however, many lack specificity for comprehensive treatment. NADPH oxidases are an attractive target for drug development for the treatment of hypertension. For example, the drug Apocynin has demonstrated potential as a NOX inhibitor in hypertension [15]. Studies confirm that the administration of Apocynin inhibits superoxide production, preserves endothelial function, and decreases systolic blood pressure in animal models [15]. The extent of the anti-hypertensive effects of Apocynin requires further study [15]. The development of an effective, specific NOX inhibitor for hypertension in human use needs further attention.

### 2.5. NOS Uncoupling Contributing to Hypertension

Under normal physiological conditions, Nitric oxide (NO), a lipophilic gas, serves several functions, all vital for the proper health of the cardiovascular system. These functions include maintaining vascular tone such as with the endothelial-derived-relaxing factor (EDRF), the prevention of endothelial dysfunction, and the modulation of endothelial–platelet interactions [16]. NO is produced by the enzyme, nitric oxide synthase (NOS), of which there are three isoforms: eNOS (endothelial NOS), iNOS (inducible NOS), and nNOS (neuronal NOS) [6]. During the oxidative stress state associated with hypertension, the decrease/misregulation of NO bioavailability contributes to the pathology of the condition. With the increase in ROS, eNOS becomes upregulated; however, this upregulation does not produce enough NO to compensate for deleterious changes [16]. Under normal conditions, the cofactor tetrahydrobiopterin (BH4) aids eNOS catalyzation of L-arginine to L-citrulline and the production of NO. During hypertension, BH4 levels are insufficient, shifting eNOS to produce superoxide [16]. Superoxide converts BH4 to BH2, thus destabilizing and uncoupling NOS, leading to impairments in NO production [6,16]. The superoxide produced by the overactivity of NADPH oxidase can affect NO production and bioavailability by eNOS [16]. This causes decreased vascular tone, platelet aggregation, and endothelial dysfunction, all of which are main causative factors in hypertension.

### 2.6. Genetic and Environmental Factors in Hypertension

Several genetic and environmental factors play a role in the development of hypertension. Genetically, studies have confirmed that polymorphisms in genes PRKG1 rs1904694, HSD3B1 rs2236780, and HSD3B1 rs6203 are associated with systolic blood pressure [17]. Diastolic blood pressure is associated with genes CYP11B2 rs1799998 and NEDD4L rs4149601 [17]. One gene of particular interest, LSS rs2254524, when presented as a missense variant, is highly linked to systolic and diastolic blood pressure. Noted increases in both pressure measurements occur in carriers of one or two LSS A alleles [17]. Due to the multifactorial etiological nature of the development of hypertension, identifying the root cause of the condition is complex. Theoretically, it has been suggested that many genes, which have been altered due to environmental stressors, contribute to the development and progression of hypertension [18].

Two influential environmental factors contributing to the development of hypertension are air pollution and traffic noise [19]. Prolonged exposure to particulate matter in polluted air can activate the sympathoadrenal system, autonomic nervous system, pro-inflammatory pathways, and can induce endothelial dysfunction [19]. Exposure to the loudness associated with noise pollution, acute or chronic, unfavorably activates autonomic and endocrine systems. It is well known that improper activation of these systems significantly contributes to the development and progression of hypertension.

Furthermore, it is essential to mention the impact of poor diet on the development of hypertension. The prevalence of ultra-high-processed, high-fat, high-cholesterol foods is at an all-time high in the human diet. Chronic overconsumption of these foods can lead to obesity, where there is a characterized persistent overactivation of the renin–angiotensin system accompanied with prolonged activation of the sympathetic nervous system [20]. These prolonged physiological changes can lead to the development of hypertension. Lastly, the main source of excess sodium in the human diet is derived from processed and ultra-high-processed foods [21]. The exact mechanism of how increased sodium leads to high blood pressure needs to be further elucidated. This relationship produces an array of disadvantageous effects including detrimental effects on the vascular walls, increased peripheral resistance, endothelial dysfunction/stiffness, and dysfunctional neuro-hormonal pathways [21]. The physiological consequences due to long-term poor dietary choices are attributed to the development of hypertension.

A thorough understanding of hypertension is essential for the development of effective interventions for treatment. Sesn2, a highly conserved endogenous protein, has gained a heavy amount of traction in research over the past 5 years. Sesn2 has the structural and functional capabilities to target the previously described factors and serve as a therapeutic target in the treatment of hypertension.

## 3. Sestrin2

### 3.1. Background and Function

The Sestrin (SESN) family of proteins is comprised of three members Sesn1, Sens2, and Sesn3, and is expressed by three coding genes: SESN1, SESN2, and SESN3 [22,23,24]. These proteins are characterized as stress-inducible-metabolic regulators and are upregulated during high-stress conditions such as DNA damage and oxidative stress [24]. Of the three members of the family, Sesn2 is the main protein responsible for responding to increases in oxidative stress [23]. Sesn2 also serves a variety of beneficial functions. Sesn2 can activate adenosine monophosphate-activated protein kinase (AMPK), thus inhibiting the mammalian target of rapamycin (mTOR) [22]. Further, Sesn2 can coordinate various antioxidant pathways, act directly as an antioxidant enzyme, and produce anti-inflammatory effects [22,23]. These unique properties can combat the etiology of hypertension, making Sesn2 an excellent target in the development of therapeutic treatment plans for hypertension.

### 3.2. Structure

Sesn2 is a fully alpha helical globular monomeric protein with a molecular weight of approximately 55 kDa [25]. Three main substructures are of note with Sesn2: an N-terminal domain (SesnA) and a C-terminal domain (SesnB), which are connected by a linker region (SesnC) [23,25]. SesnA, a small area of residues in the N-terminal domain, is the location of Sesn2′s antioxidant enzyme activity, acting as an alkyl hydroperoxide reductase [23,25]. Sesn2 can interact with leucine, which structurally occurs near the SesnB region of residues Asp 406 and Asp 407 [23,25]. The effective interaction with leucine is of utmost importance, as leucine is necessary to further induce AMPK activation, reducing the activity of mTORC1, and is also involved in many biological processes of Sesn2 functioning [25]. Continuing, we will further discuss in this review the axiomatic relationships of various signaling pathways of Sesn2, including nuclear factor erythroid 2-related factor 2 (Nrf2), AMPK/mTORC, and Ang II in relation to hypertension. With insight into the regulatory properties of Sesn2 in these various signaling pathways, we will then discuss how Sesn2 can be utilized as a treatment option for hypertension.

### 3.3. Sesn2 and Nrf2

**Figure 2:**
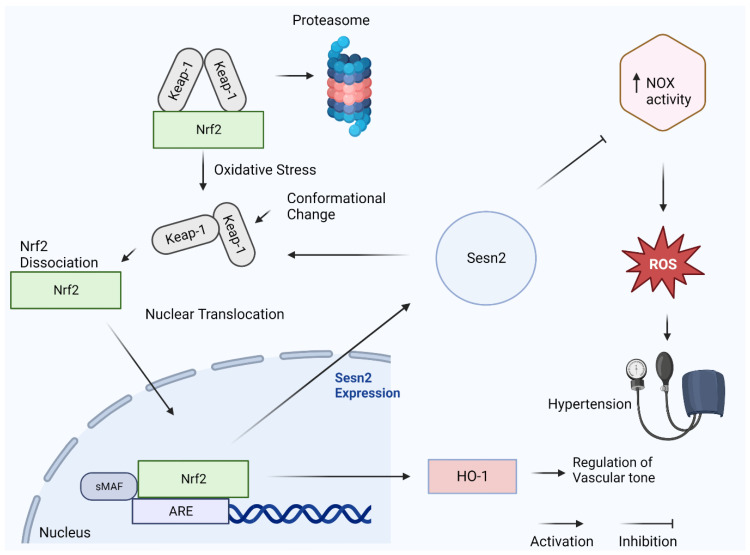
Nrf2/Sesn2/Nox axis and hypertension (created with BioRender.com, accessed on 11 May 2023).

Nuclear factor erythroid 2 (Nrf2) is a transcription factor involved in the expression of antioxidant proteins [26]. Nrf2 is found in the cytoplasm during normal physiological conditions bound by Keap-1 and undergoes normal proteasomal degradation to maintain homeostatic levels [23]. Under oxidative stress conditions, Nrf2 travels to the nucleus where it binds to the antioxidant response element (ARE) region of antioxidant genes [27]. This gene region promotes the production of several antioxidant proteins including Sesn2 (Figure 2) [27]. Nrf2 also contributes to the expression of the anti-hypertensive enzyme heme-oxygenase 1 (HO-1). Increased expression of HO-1 degrades heme, producing carbon monoxide, bilirubin, and iron [28]. The carbon monoxide created is a known vasodilator. Proper vasodilatory effects can aid in the regulation of vascular tone, a hallmark characteristic misregulated in hypertension.

Nrf2 and Sesn2 interact in a positive feedback loop manner [23] As mentioned, Nrf2 activation induces the production of Sesn2. After Sens2 is produced, it can act as a scaffold protein, promoting the degradation of Keap-1, thus leading to more Nrf2 activation and further promoting the production of various antioxidants [23]. As previously mentioned, NOX isoforms contribute to the development of hypertension via the overproduction of ROS. The Nrf2/Sesn2 axis is highly affiliated with changes in the expression of the NOX family isoforms [23]. Proper management of overactive NOX is essential to mitigate the ROS produced during the development of hypertension. NOX2 elevation is directly associated with increases in blood pressure. Studies show that with Nrf2 deficiency, NOX2 upregulation is observed, linking the potential of the Nrf2/Sesn2 axis to hypertension [23]. Also, as previously described, NOX4 has vasculo-protective effects. NOX4 upregulation is associated with increases in Nrf2 [23]. Based on these findings, Nrf2 agonists such as curcumin and sulforaphane have been the focus of current research. Due to the complexity and the specific nature of Nrf2 activation, an effective agonist has yet to be developed to target hypertension. The Sesn2/Nrf2/NOX family axis is a high-profile target in the development of a treatment option for hypertension and warrants further investigation.

### 3.4. Sesn2 and Angiotensin II

**Figure 3:**
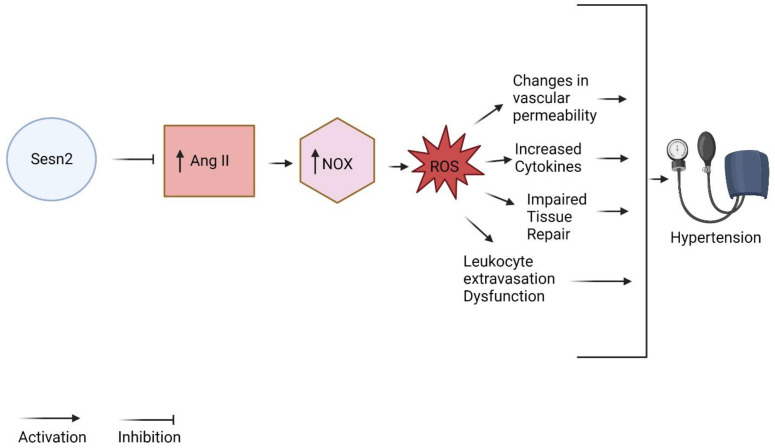
Sesn2/Ang II/Nox axis in hypertension (created with BioRender.com, accessed on 15 May 2023).

The peptide, Ang II, is a major component in the renin–angiotensin system (RAS) [8]. The RAS is responsible for the regulation of key factors seen in hypertension, such as electrolyte balance, the regulation of blood volume, and vascular resistance [29]. Overactivation/misregulation of the RAS, including Ang II, can result in a multitude of diseases including hypertension. In hypertension, overactive Ang II can activate NADPH oxidase and produce increased amounts of ROS [8]. The ROS produced due to the overactivation of Ang II produces pro-inflammatory effects. These effects include alterations in vascular permeability, cytokine production, misregulation of tissue repair, and dysfunction of leukocyte extravasation [8,30]. Proper management of the overactive Ang II exhibited in hypertension by Sesn2 can lead to a decrease in the ROS produced by NADPH oxidase (Figure 3). Targeting Ang II could therefore reduce the major pro-inflammatory progression of hypertension and produce a reduction in pathophysiology. Sesn2 is well known to interact with Ang II. Specifically, Ang II can increase the proliferation of cardiac fibroblasts and increase the production of collagen [23]. Multiple studies have demonstrated Sesn2′s effects on Ang II in ameliorating these effects [23]. Studies involving human umbilical vein cells show Sesn2 decreasing Ang II-induced ROS production [23]. Also, silencing Sens2 resulted in worsened cell viability and increased Ang II activity [23]. Further analysis of the interaction between Sesn2 and Ang II is needed to observe the potential advantageous downstream effects of the axis on the pathophysiology of hypertension.

### 3.5. Sesn2, AMPK, and mTOR

**Figure 4:**
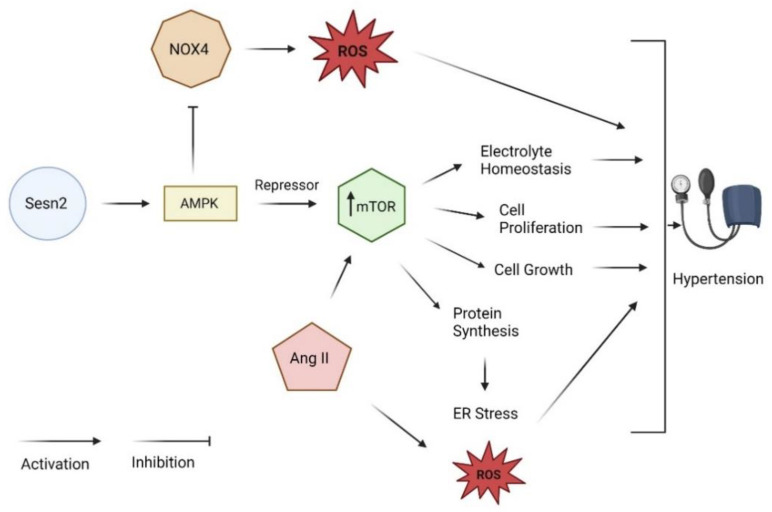
Sesn2/AMPK/mTOR in hypertension (created with BioRender.com, accessed on 17 May 2023).

Two protein complexes, mTORC1 and mTORC2 share the same serine/threonine protein kinase catalytic subunit, mTOR [24,31]. These proteins participate in a range of functions including electrolyte homeostasis, cell growth, cell proliferation, immune system regulation, and activation of protein synthesis [24,31]. Specifically, mTORC1 serves regulatory purposes as it acts in response to oxidative stress and changes in energy levels [24]. The precise function of mTORC2 needs further examination. However, it has been reported to play a role in the regulation of renal tubular sodium and potassium transport [31]. Persistent stimulation of mTORC1 serves as a signaling pathway in the development of fibrosis and collagen disposition, contributing to the development of hypertension [32]. The increase in Ang II can also activate mTORC1; however, knowledge of this pathway is understudied [32]. Chronic activation of mTORC1 can lead to the overactivation of protein synthesis, thus generating ER stress and the production of ROS. Sesn2 can interact and mitigate mTORC1 via disruption of the Ras homolog enriched in the brain (Rheb) and Ras-related GTPase A/B axis, two of the GTPases responsible for the activation of mTORC1 [23]. An upstream regulator of mTOR, AMPK can be activated by Sesn2 to mitigate these effects produced by the chronic stimulation of mTOR (Figure 4) [24]. Further, AMPK activation by Sesn2 can influence overactive NOX4, decreasing the ROS production exhibited in hypertension [24].

### 3.6. Sesn2 Protein as a Therapeutic Modality for the Treatment of Hypertension

The details mentioned in this review indicate the potential of Sesn2 as a therapeutic target for hypertension. A comprehensive understanding of the nature of Sesn2 and its upstream/downstream effects can give insight into its therapeutic potential. Currently, there are no known direct pharmacological correlates to the mechanism of Sesn2. A Sesn2 mimetic could be developed where the small molecule’s active sites have more affinity for the downstream targets, contributing to specificity. Furthermore, Sesn2 activators and inducers should be further investigated, as these could induce Sesn2 in non-oxidative stress conditions. This could prove advantageous in the early, or prophylactic treatment of disease.

As mentioned, an activator of Sesn2 is Nrf2. Nrf2 agonists such as sulforaphane and curcumin are under heavy investigation for their downstream antioxidant protein activation effects. An effective Nrf2 agonist has yet to be developed due to a lack of specificity. A delicate balance in ROS scavenging capabilities is of utmost importance. A drop in ROS can contribute to alleviating the progression of disease. However, drastic ROS scavenging levels or those that lack specificity could lead to the development of unwanted disease. For example, ROS are used in immune function. The hyper-targeting or removal of ROS function could lead to dysfunction of the immune system, allowing opportunities for pathogens to induce disease. The development of a high-specificity Nrf2 agonist could activate Sesn2 in non-oxidative or low-oxidative stress conditions, such as early or pre-hypertension. The Nrf2/Sesn2 pathway could lead to a viable early treatment modality for hypertension and warrants further examination.

Lastly, adeno-associated virus (AAV) vectors, an effective method for gene therapy delivery, could be utilized to favorably manipulate Sesn2 in hypertension. AAV vectors can replace genes, silence genes, edit genes, and knock-in genes [33]. The use of the vectors could potentially increase Sesn2 expression to counteract the high amount of ROS production/oxidative stress exhibited in hypertension. Also, AAV vectors could favorably edit the gene for Sesn2, producing a Sesn2 protein whose active sites have a highly specific and effective affinity for their respective downstream targets such as NADPH oxidase and Ang II.

### 3.7. Conclusions

Hypertension is a condition producing detrimental health effects across the globe. Almost all hypertension cases are uncontrolled. The adverse health effects, mortality rate, and societal impact of hypertension place the development of an effective treatment plan in urgent demand. Sesn2, a highly conserved, stress-inducible protein, has the potential to serve as a therapeutic target in treating hypertension. Sesn2 possesses antioxidant properties and can interact with various signaling pathways involved in hypertension. Further exploration is needed on the antioxidant properties and signaling pathway interactions of Sesn2 in hypertension. Newly discovered findings could give rise to the development of a highly specific and effective treatment protocol involving Sesn2 to ameliorate the effects of hypertension.

## Data Availability

Not applicable.

## Abbreviations

AAR	Area at risk
AAV	Adeno-associated virus
AMPK	Adenosine monophosphate-activated protein kinase
ANG II	Angiotensin 2
BH4	Tetrahydrobiopterin
EDRF	Endothelial-derived relaxing factor
eNOS	Endothelial nitric oxide synthase
ER	Endoplasmic Reticulum
ET-1	Endothelin 1
mTOR	Mammalian target of rapamycin
NADPH	Nicotinamide adenine dinucleotide phosphate
NO	Nitric oxide
NOS	Nitric oxide synthase
NOX	NADPH oxidase
Nrf2	Nuclear factor erythroid 2
RAS	Renin–angiotensin system
ROS	Reactive oxygen species
Sesn2	Sestrin2
TNF	Tumor necrosis factor

## References

[1] Oparil S., Acelajado M.C., Bakris G.L., Berlowitz D.R., Cífková R., Dominiczak A.F., Grassi G., Jordan J., Poulter N.R., Rodgers A. (2018). Hypertension (Primer). Nat. Rev. Dis. Prim..

[2] Giles T.D., Berk B.C., Black H.R., Cohn J.N., Kostis J.B., Izzo J.L., Weber M.A. (2005). Expanding the definition and classification of hypertension. J. Clin. Hypertens..

[3] MacLeod K.E., Ye Z., Donald B., Wang G. (2022). A Literature Review of Productivity Loss Associated with Hypertension in the United States. Popul. Health Manag..

[4] Dosh S.A. (2001). The diagnosis of essential and secondary hypertension in adults. J. Fam. Pract..

[5] Chiong J.R., Aronow W.S., Khan I.A., Nair C.K., Vijayaraghavan K., Dart R.A., Behrenbeck T.R., Geraci S.A. (2008). Secondary hypertension: Current diagnosis and treatment. Int. J. Cardiol..

[6] Griendling K.K., Camargo L.L., Rios F.J., Alves-Lopes R., Montezano A.C., Touyz R.M. (2021). Oxidative Stress and Hypertension. Circ. Res..

[7] Paravicini T.M., Touyz R.M. (2008). NADPH oxidases, reactive oxygen species, and hypertension: Clinical implications and therapeutic possibilities. Diabetes Care.

[8] Masi S., Uliana M., Virdis A. (2019). Angiotensin II and vascular damage in hypertension: Role of oxidative stress and sympathetic activation. Vascul. Pharmacol..

[9] Dikalov S.I., Ungvari Z. (2013). Role of mitochondrial oxidative stress in hypertension. Am. J. Physiol. Heart Circ. Physiol..

[10] Widlansky M.E., Gutterman D.D. (2011). Regulation of endothelial function by mitochondrial reactive oxygen species. Antioxid. Redox Signal..

[11] Halperin L., Jung J., Michalak M. (2014). The many functions of the endoplasmic reticulum chaperones and folding enzymes. IUBMB Life.

[12] Young C.N. (2017). Endoplasmic reticulum stress in the pathogenesis of hypertension. Exp. Physiol..

[13] Zhou Y., Zhao L., Zhang Z., Lu X. (2015). Protective Effect of Enalapril against Methionine-Enriched Diet-Induced Hypertension: Role of Endoplasmic Reticulum and Oxidative Stress. Biomed. Res. Int..

[14] Konior A., Schramm A., Czesnikiewicz-Guzik M., Guzik T.J., Urner S., Ho F., Jha J.C., Ziegler D., Jandeleit-Dahm K., Karki P. (2014). NADPH oxidases in vascular pathology. Antioxid. Redox Signal..

[15] Williams H.C., Griendling K.K. (2007). NADPH oxidase inhibitors: New antihypertensive agents?. J. Cardiovasc. Pharmacol..

[16] Jin R.C., Loscalzo J. (2010). Vascular Nitric Oxide: Formation and Function. J. Blood Med..

[17] Bigazzi R., Zagato L., Lanzani C., Fontana S., Messaggio E., Carpini S.D., Citterio L., Simonini M., Brioni E., Magnaghi C. (2020). Hypertension in High School Students: Genetic and Environmental Factors. Hypertension.

[18] Kunes J., Zicha J. (2009). The interaction of genetic and environmental factors in the etiology of hypertension. Physiol. Res..

[19] Bruno R.M., Di Pilla M., Ancona C., Sørensen M., Gesi M., Taddei S., Munzel T., Virdis A. (2017). Environmental Factors and Hypertension. Curr. Pharm. Des..

[20] Savica V., Bellinghieri G., Kopple J.D. (2010). The effect of nutrition on blood pressure. Annu. Rev. Nutr..

[21] Juul F., Vaidean G., Parekh N. (2021). Ultra-processed Foods and Cardiovascular Diseases: Potential Mechanisms of Action. Adv. Nutr..

[22] Liu Y., Du X., Huang Z., Zheng Y., Quan N. (2020). Sestrin 2 controls the cardiovascular aging process via an integrated network of signaling pathways. Ageing Res. Rev..

[23] Liu Y., Li M., Du X., Huang Z., Quan N. (2021). Sestrin 2, a potential star of antioxidant stress in cardiovascular diseases. Free Radic. Biol. Med..

[24] Pasha M., Eid A.H., Eid A.A., Gorin Y., Munusamy S. (2017). Sestrin2 as a Novel Biomarker and Therapeutic Target for Various Diseases. Oxidative Med. Cell. Longev..

[25] Gao A., Li F., Zhou Q., Chen L. (2020). Sestrin2 as a potential therapeutic target for cardiovascular diseases. Pharmacol. Res..

[26] Shin B.Y., Jin S.H., Cho I.J., Ki S.H. (2012). Nrf2-ARE pathway regulates induction of Sestrin-2 expression. Free Radic. Biol. Med..

[27] Bhakkiyalakshmi E., Sireesh D., Ramkumar K.M. (2018). Redox Sensitive Transcription via Nrf2-Keap1 in Suppression of Inflammation. Immun. Inflamm. Health Dis..

[28] Chen Y.-H., Yet S.-F., Perrella M.A. (2003). Role of Heme Oxygenase-1 in the Regulation of Blood Pressure and Cardiac Function. Exp. Biol. Med..

[29] Fountain J.H., Kaur J., Lappin S.L. (2023). Physiology, Renin Angiotensin System.

[30] Touyz R.M. (2005). Molecular and cellular mechanisms in vascular injury in hypertension: Role of angiotensin II—Editorial review. Curr. Opin. Nephrol. Hypertens..

[31] Kumar V., Evans L.C., Kurth T., Yang C., Wollner C., Nasci V., Zheleznova N.N., Bukowy J., Dayton A., Cowley A.W.C. (2019). Therapeutic Suppression of mTOR (Mammalian Target of Rapamycin) Signaling Prevents and Reverses Salt-Induced Hypertension and Kidney Injury in Dahl Salt-Sensitive Rats. Hypertension.

[32] Guimaraes D.A., Passos M.A.D., Rizzi E., Pinheiro L.C., Amaral J.H., Gerlach R.F., Castro M.M., Tanus-Santos J.E. (2018). Nitrite exerts antioxidant effects, inhibits the mTOR pathway and reverses hypertension-induced cardiac hypertrophy. Free Radic. Biol. Med..

[33] Wang D., Tai P.W.L., Gao G. (2019). Adeno-associated virus vector as a platform for gene therapy delivery. Nat. Rev. Drug Discov..

